# The role of ^18^FDG–PET imaging in VEXAS syndrome: a multicentric case series and a systematic review of the literature

**DOI:** 10.1007/s11739-024-03763-9

**Published:** 2024-09-09

**Authors:** Riccardo Bixio, Sara Bindoli, Andrea Morciano, Roberto Padoan, Federico Aldegheri, Francesca Mastropaolo, Eugenia Bertoldo, Denise Rotta, Matteo Appoloni, Giovanni Orsolini, Davide Gatti, Giovanni Adami, Ombretta Viapiana, Maurizio Rossini, Paolo Sfriso, Angelo Fassio

**Affiliations:** 1 Rheumatology Unit, Department of Medicine, University of Verona, P.le L.A. Scuro 10, 37134 Verona, Italy; 2 Rheumatology Unit, Department of Medicine, University of Padova, Padua, Italy; 3 Internal Medicine Department, Hospital Mater Salutis, Legnago, Verona Italy

**Keywords:** VEXAS syndrome, Myelodysplastic–myeloproliferative diseases, Positron-emission tomography, Adult-onset still's disease, Giant cell arteritis

## Abstract

VEXAS (vacuoles, E1 enzyme, X-linked, autoinflammatory, and somatic) syndrome is characterized by heterogeneous clinical manifestations. Due to the inflammatory nature of this condition, 18-FDG–PET (18-fluorodeoxyglucose–positron emission tomography) might be used to diagnose and monitor the disease. However, no data are available about the most common findings of PET imaging in this disease. For this reason, we summarised all the available reports of patients with VEXAS who underwent at least one PET scan and described 8 additional patients’ PET from our centres. Overall, we described 35 patients’ PET findings. All patients were male, with a median age of 70 years. The most frequent hypermetabolic sites on PET scans were the bone marrow (77.1%), lymph nodes (35.3%), lungs (28.6%), spleen and large vessels (22.9%), and cartilage (20%). Six patients underwent a PET scan 2.7 ± 1.5 years before VEXAS diagnosis, showing nonspecific uptake in the bone marrow. Four patients had a follow-up PET scan, showing a decrease or a disappearance of the previously identified hypermetabolic areas. In conclusion, although no specific uptake site has been found for VEXAS syndrome, PET imaging could help detect inflammatory foci that are not clinically evident. In addition, high metabolic activity in bone marrow might precede the clinical onset of the disease, shedding light on the pathogenesis of VEXAS.

**Supplementary Information:**

The online version contains supplementary material available at 10.1007/s11739-024-03763-9.

## Background

Vacuoles, E1 enzyme, X-linked, autoinflammatory, and somatic (VEXAS) syndrome is a recently identified adult-onset autoinflammatory syndrome. It is driven by a mosaic mutation of ubiquitin-like modifier activating enzyme 1 (UBA1) gene [1]. Clinical manifestations of VEXAS syndrome are heterogeneous and range from constitutional symptoms like weight loss, asthenia, and fever driven by systemic inflammation to macrocytic anemia and other cytopenias, variable skin rashes, lung involvement, orchitis, and polychondritis. There is often a co-occurrence of hematological manifestations, such as myelodysplastic syndrome (MDS). Furthermore, VEXAS syndrome has been associated with an elevated risk of myeloid malignancies and both large and small vessel vasculitis [2, 3]. Current treatments primarily rely on glucocorticoids, since common biological disease-modifying antirheumatic drugs (bDMARDs), such as anakinra and tocilizumab, which target interleukin (IL)-1 and IL-6, respectively, often prove ineffective. Although there is some promising evidence surrounding Janus kinase inhibitors (JAKi), the data remain limited [4–6].

There are no accepted diagnostic criteria for this syndrome, and diagnosis relies on finding a pathogenic UBA1 mutation in patients with hematological and inflammatory manifestations. Various sets of diagnostic clues to decide whom to test for UBA1 mutations have been developed [7]. Still, currently, none includes imaging techniques, whose role in this syndrome remains an under-researched topic. In routine clinical practice, various imaging techniques are used to deal with VEXAS organ involvement, such as chest computed tomography (CT) for lung infiltrates, angio-CT for large vessel inflammation, and Doppler ultrasound for detecting thrombotic events [8–11]. However, none of them appears specific for VEXAS syndrome. In the realm of autoinflammatory syndromes, 18-fluorodeoxyglucose (FDG)–positron emission tomography (PET), combined with either computed tomography (CT) or magnetic resonance (MR), is one of the preferred imaging method. It is typically used to identify inflammatory foci, as seen in large vessel vasculitis and other autoinflammatory syndromes, and also to detect malignancies. However, data regarding PET imaging findings in VEXAS syndrome are largely confined to isolated case reports. To the best of our knowledge, no comprehensive analysis assessing the utility of PET in this syndrome has been conducted. The present study aims to describe a series of VEXAS patients who underwent PET imaging, and to conduct a systematic review of existing PET reports on this rare syndrome. Our aim is to assess the most prevalent findings and evaluate the potential utility of PET in diagnosing and monitoring VEXAS.

## Methods

We described clinical cases of outpatients diagnosed with VEXAS syndrome from both the Rheumatology Section of the University of Verona and of the University of Padova between 1st January 2021 and 31st December 2023. Each patient had undergone at least one ^18^FDG–PET/CT or ^18^FDG–PET/MR scan. Case details were extracted from the patients’ electronic records and described according to CARE guidelines [12]. Every patient provided written informed consent, as approved by the local Hospital Trust Ethic Committee (protocol 1483CESC). To retrieve further cases of patients with VEXAS syndrome in which at least one PET imaging report was available, we designed a systematic review of the literature. The protocol of the systematic review of the literature was registered on “International Prospective Register of Systematic Reviews” (PROSPERO), registration code CRD42024511598. We included papers that described at least one patient with a VEXAS diagnosis confirmed by gene mutation and sufficient 18-FDG–PET findings. The review of the literature was conducted following the 2020 “Preferred Reporting Items for Systematic Reviews and Meta-Analyses” (PRISMA 2020) guidelines [13], using the following databases: MEDLINE/PubMed via OVID, EMBASE via OVID and Cochrane Library via Cochrane Central. Search included articles from inception of each database to January 17th, 2024. Search query and PRISMA checklist are included in Supplementary Data S1 and Supplementary Material (PRISMA checklist). An additional unsystematic manual search and citation matching was also conducted to identify potential articles. The search included randomized controlled trials, observational studies, case series, and case reports with a sample size of one or more subjects; editorals, narrative reviews, commentaries, and secondary articles were excluded. After duplicate removals, two authors (RB and AM) independently reviewed the articles to determine their suitability based on the inclusion/exclusion criteria. A risk of bias assessment was performed by two reviewers (RB and AM) using the Joanna Briggs Institute (JBI) tool for case reports, with reports scoring ≥ 4 considered “high quality” and < 4 “low quality” [14, 15]. Rating has been performed for each case described. In case of disagreement, a third reviewer (AF) assessed the report separately. Bias analysis results are reported in Supplementary Table S1). When available, for both our cases and those retrieved from the literature, the following data were included in descriptive analyses: gender, age at the time of the ^18^FDG–PET/CT, UBA1 mutation, clinical manifestations, PET findings, and details of any previous or subsequent PET scans. Subsequently, a descriptive statistical analysis was conducted on the totality of the patients, both retrieved from the literature and described in the present paper, using IBM SPSS Statistics for Windows, version 26 (IBM Corp., Armonk, N.Y., USA). The main findings are summarised in tables, graphical summary and thorough text as narrative review, both as overall description and in separate subgroup description for patients whose PET scan predated the VEXAS syndrome diagnosis.

## Results

### Case series

#### Case 1

**Fig. 1:**
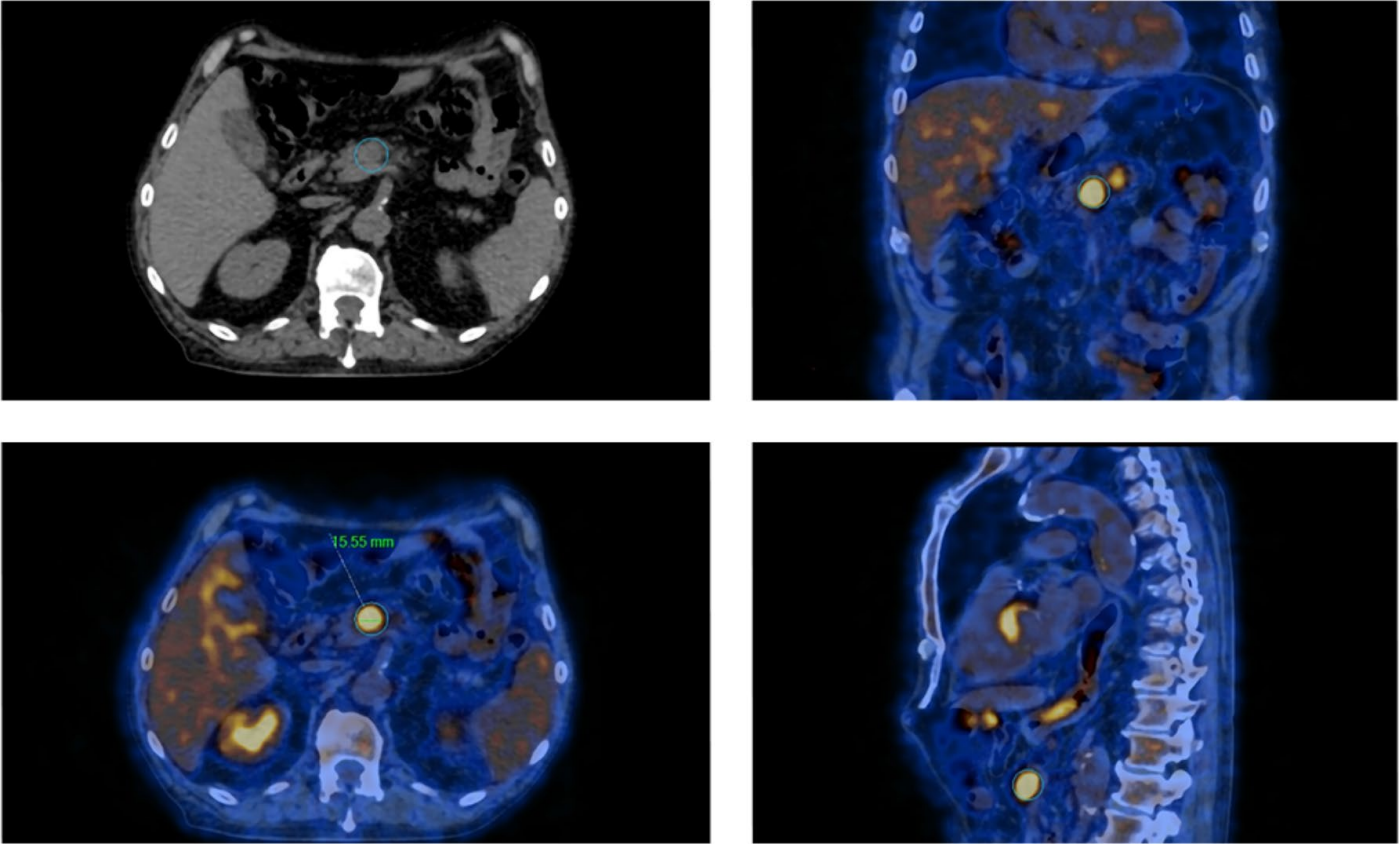
Case 1, 18-FDG–PET/CT scans of pancreatic and lymph node hypermetabolism. *18-FDG* 18 fluorodeoxyglucose, *PET/CT* positron emitting tomography/computed tomography

**Fig. 2:**
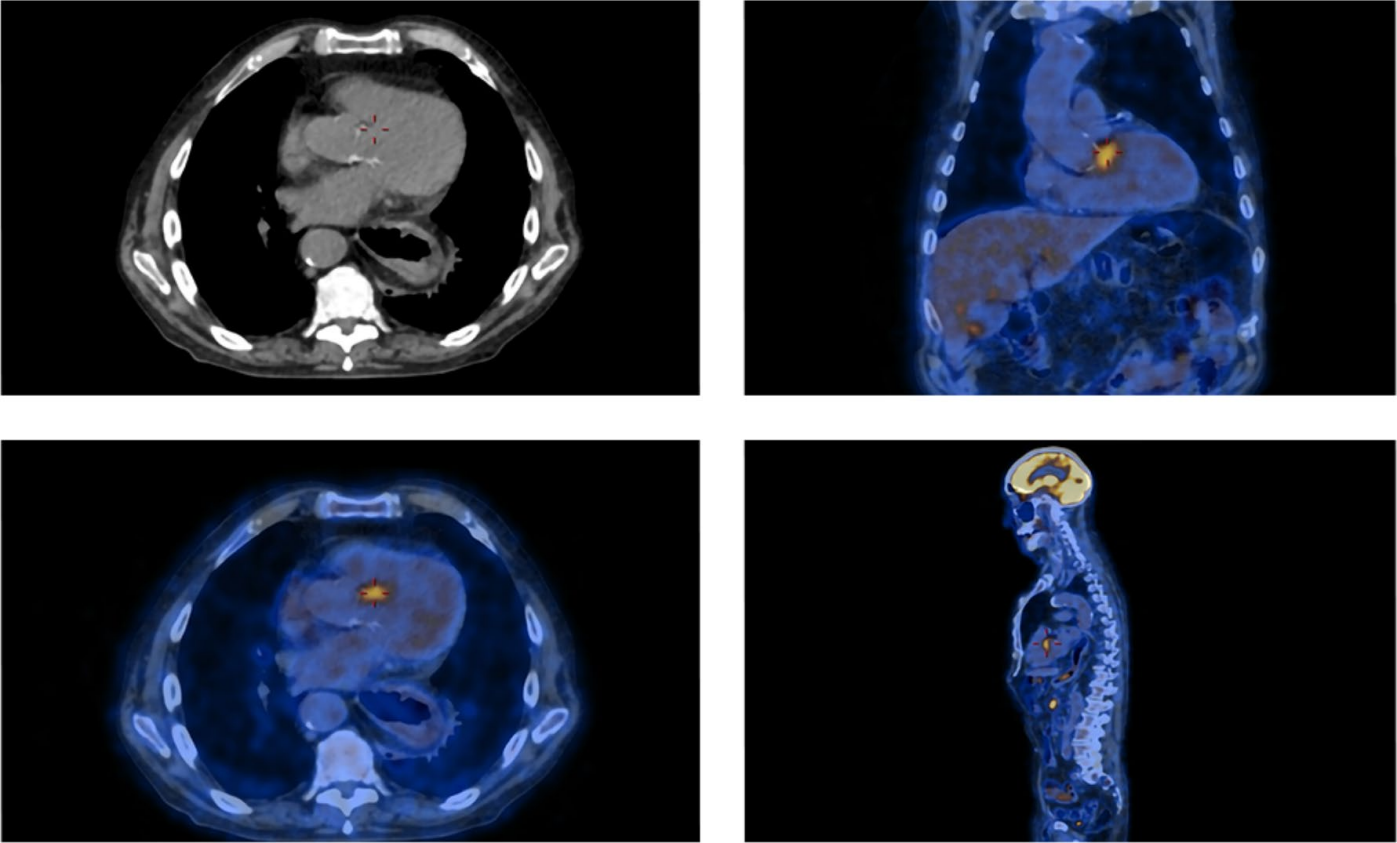
Case 1, 18-FDG–PET/CT scans of aortic valve hypermetabolism. *18-FDG* 18 fluorodeoxyglucose, *PET/CT* positron emitting tomography/computed tomography

An 80-year-old man presented with a 7-month history of fever unresponsive to antibiotic therapy, weight loss, periorbital oedema, deep venous thrombosis (DVT), anaemia, hydrocele, and elevated acute phase reactants. As part of his diagnostic work-up, he underwent an ^18^FDG–PET/CT scan, revealing diffuse metabolic activity in the bone marrow. A bone marrow biopsy ruled out hematologic malignancies but identified vacuoles in myeloid precursor cells. Genetic testing for adult-onset autoinflammatory syndromes was performed and revealed UBA1 c,121 A > G p.(Met41Val) mutation. Two years later, the patient reported a temporal headache, a notable elevation in c-reactive protein (CRP—80 mg/L) and ferritin (2533 ug/L). A temporal artery ultrasound suggested temporal arteritis, prompting an increase in glucocorticoid therapy to prednisone 1 mg/kg/day. This approach quickly improved symptoms and reduced inflammatory markers. To investigate the possibility of large vessel vasculitis, a second ^18^FDG–PET/CT was conducted. While metabolic activity of large vessels resulted normal, the PET scan showed a focal hypermetabolic area in the pancreatic isthmus (1.5 cm) with a standardised uptake value (SUV) of 9.8, a satellite area (< 1 cm–SUV 5.3) suggestive of a lymph node (Fig. 1), and significant hypermetabolism (SUV 6.7) at the aortic valve consistent with endocarditis (Fig. 2). A transesophageal echocardiography (TEE) identified a small (2 mm) vegetation on the aortic valve. Intravenous antibiotic therapy with vancomycin was started, while ongoing glucocorticoid and anticoagulant treatments were continued. All microbiological tests returned negative results. To further examine the pancreatic lesion, a contrast-enhanced CT scan was performed which identified early fibro-adipogenic changes in the pancreas and a small hypodense area (13 mm) in the pancreatic isthmus, which appeared hypoperfused in all contrast phases. Subsequent imaging, utilizing endoscopic ultrasound (EUS) and contrast-enhanced MR imaging, did not detect this lesion, and a biopsy was not possible. All oncologic markers were within normal limits. A follow-up PET/CT, performed 4 weeks later, indicated a reduction in the size and metabolic activity of the main pancreatic lesion (1 cm, SUV 5.3), with the secondary lesion no longer visible. In addition, the valvular uptake was also reduced (SUV 4.6).

#### Case 2

An 85-year-old man was admitted to the inpatient clinic due to symptoms of relapsing polychondritis, fever, skin vasculitis lesion, and erythema nodosum-like lesions. To rule out systemic vasculitis, an 18-FDG–PET/CT scan was conducted. The imaging revealed diffuse and intense metabolic activity in the bone marrow, with especially high uptake in the right iliac region (SUV 9.6). Other hypermetabolic areas were identified in the spleen (SUV 5.3), right ear and nasal region (current localization of chondritis), the origin of the right subclavian artery (SUV 4.6), left lower lung lobe, and cervical lymph nodes. Treatment with glucocorticoids at a dose of 1 mg/kg/day was started, leading to a swift resolution of clinical symptoms and a reduction in systemic inflammatory markers. Although the bone marrow biopsy and hematologic testing were inconclusive, genetic testing for adult-onset autoinflammatory diseases was carried out, revealing a pathogenetic UBA1 mutation c.122T > C p.(Met41Thr), confirming the diagnosis of VEXAS syndrome.

#### Case 3

A 75-year-old man was followed at the outpatient due to a 4-year history of elevated acute phase reactants, thrombocytopenia, fever, recurrent thrombophlebitis, and erythema nodosum. As part of this diagnostic evaluation, a PET–CT scan was conducted, revealing only a diffuse bone marrow hypermetabolism. An initial diagnosis of unclassified autoinflammatory fever was made, leading to treatment with glucocorticoids, anakinra (which was discontinued due to lymphopenia), and tocilizumab (discontinued due to neutropenia and infections). Given the persistent thrombocytopenia, he later underwent a bone marrow examination. This showed vacuoles in the myeloid precursor cells. Consequently, UBA1 genetic testing was conducted, revealing a p.Met41Val mutation. In conjunction with the clinical presentation, these findings confirmed a diagnosis of VEXAS syndrome.

#### Case 4

In 2019, a 60-year-old man was admitted to the hospital due to recurrent fever, elevated inflammatory markers (including CRP, serum amyloid A and ferritin), macrocytic anaemia, widespread arthralgias and auricular chondritis. In addition, he presented with a skin rash consistent with Sweet’s syndrome. Throughout his hospital stay, he underwent several laboratory tests and diagnostic procedures, which ruled out infections or haematological malignancies. A bone marrow biopsy revealed vacuolization in myeloid precursor cells. At that time, UBA1 genetic testing was not yet available, so a Sanger sequencing of the MEFV gene was conducted, without evidence of significant mutations on exon 10. A subsequent 18F-FDG–PET/CT scan detected uptakes in cervical lymph nodes (SUV max 6.31). Despite several immunosuppressive treatments, the patient did not observe any significant benefit. Given the suspicion of VEXAS syndrome, UBA1 genetic analysis was carried out in 2021, which detected the p.Met41Thr mutation. The patient was then treated with upadacitinib 15 mg/day, leading to a significant remission in clinical symptoms and a decrease in inflammatory markers. However, after 6 months of treatment, he developed a severe lung infection. He was readmitted to the hospital, but unfortunately died a few weeks later, even after receiving adequate antimicrobial treatments.

#### Case 5

An 85-year-old man was admitted to the hospital with suspected polymyalgia rheumatica due to symptoms of fever, muscle pain and stiffness, particularly in the shoulders, hips, and upper arms. An elevated CRP was also noted. His medical history revealed prior deep vein thrombosis in the lower limbs, for which he was on oral anticoagulants, and an episode of crystal-induced arthritis in his left wrist. To rule out potential malignancies, he underwent an ^18^F-FDG–PET/CT scan, which detected diffuse uptakes in lymph nodes (paratracheal region, aortopulmonary window, subcarinal area, hilas, and left axillary). In addition, non-specific uptakes were observed at the base of the right lung, right humeral scapula, right carpal, and the periprosthetic tissues of the right hip and of the left knee. Specific SUV values were not provided. Subsequently, the patient started with glucocorticoids and methotrexate at 15 mg/week. This regimen only provided partial relief. Six months later, he developed chondritis in his left ear, and his CRP levels remained elevated despite ongoing treatment. A follow-up PET–CT indicated consistent uptakes in the previously identified regions. Due to the potential of VEXAS syndrome, genetic testing was pursued, uncovering the c.118-1G > C mutation in the UBA1 gene. He started a JAK-1 selective inhibitor, filgotinib 200 mg/day, with rapidly alleviated both his clinical and laboratory markers.

#### Case 6

In 2017, a 68-year-old man was admitted to the hospital due to hyperpyrexia, arthritis, sensitive neuropathy, widespread vasculitic lesions, polyserositis and chondritis affecting both the ear and nose. A PET–CT scan identified areas of non-uniform hypermetabolism in the thigh muscles and pelvis. Metabolic activity was also detected in the right upper limb and bone marrow. He was diagnosed with recurrent polychondritis and, from 2017 until the present, was treated with varying doses of glucocorticoids, intravenous immunoglobulins, anakinra, colchicine, and ultimately methotrexate (up to 20 mg/week). These treatments provided only temporary relief. Due to persistently elevated CRP levels and the suspicion of VEXAS syndrome, genetic testing was conducted. The results revealed a p.Met41Leu mutation in the UBA1 gene, confirming the diagnosis of VEXAS syndrome.

#### Case 7

An 82-year-old man was admitted to the hospital due to symptoms resembling chronic urticaria and fever. Consequently, he was treated with several therapies including glucocorticoids, TNF-alpha inhibitors, and the anti-IgE omalizumab. Later, he developed additional complications, including pleural effusion, peripheral neuropathy, arthritis, lower limb edemas, right scleritis, and chondritis affecting both the ear and nose. A ^18^F-FDG–PET–CT scan revealed increased uptake in both upper and lower limbs as well as in the though specific SUV values and the exact vessels involved were not specified. The bone marrow biopsy did not provide a conclusive diagnosis. However, due to the persistent fever, inflammation, and macrocytic anaemia, he underwent genetic testing. This revealed the p.Met41Leu mutation in the UBA1 gene, confirming suspicions of VEXAS syndrome. The patient was then prescribed glucocorticoids, with a dosage ranging between 12.5 and 10 mg/day, which effectively managed his condition.

#### Case 8

**Fig. 3:**
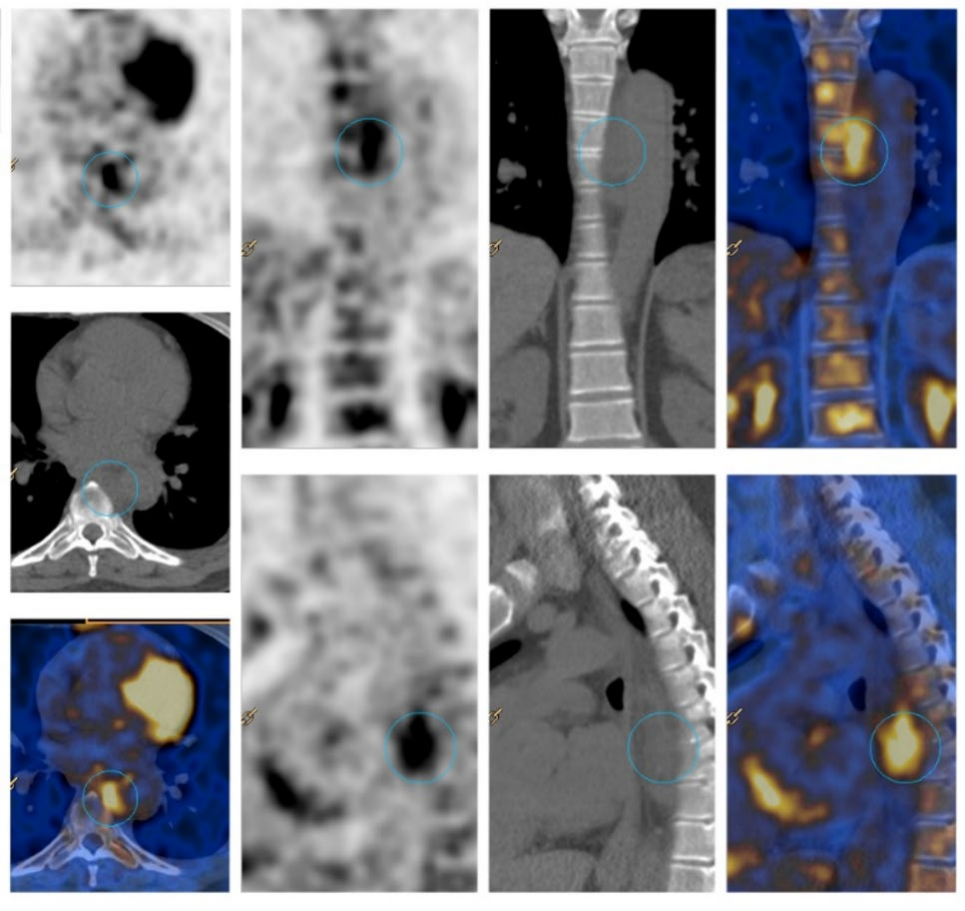
Case 8, 18-FDG–PET/CT scans of para-aortic infiltrative tissue at the level of the thoracic aorta and bone marrow hypermetabolism. *18-FDG* 18 fluorodeoxyglucose, *PET/CT* positron emitting tomography/computed tomography

A 51-year-old man was evaluated at our outpatient clinic due to persistently elevated acute phase reactants associated with a history of deep and superficial vein thrombosis of both superior and inferior extremities, for which he was on oral anticoagulants. His past medical history revealed a myelodysplastic syndrome and a prior episode of preseptal eye cellulitis. Plasmatic IgG-4 were increased (254 mg/dl ULN 104 mg/dl), suggesting a possible IgG-4-related disease. An 18F-FDG–PET–CT scan was performed, which identified a para-aortic infiltrative tissue near the thoracic aorta with increased uptake (SUV 6.7) and a diffuse bone marrow hypermetabolism (Fig. 3). In addition, the patient developed polychondritis and panniculitis. For these findings, the patient was treated with glucocorticoids with a prompt resolution of the symptoms. A contrast-enhanced CT scan was then performed to characterise better FDG–PET findings, which revealed a reduction in both dimensions and hyperintensity of the para-aortic infiltrative tissue previously identified, and the follow-up angio-MRI performed after 3 months of glucocorticoid therapy showed complete regression of the para-aortic tissue. Following the suspicion of VEXAS syndrome, genetic testing was performed, revealing c.122 T > C p.(Met41Thr) mutation in the UBA1 gene, confirming the diagnosis.

### Systematic review of the literature summary

From the database search (Flowchart, Supplementary materials, Figure S1), a total of 40 articles were found after duplicates elimination. After the screening, a total of 23 papers were deemed suitable for inclusions [6, 16–37]. Of them, 22 were single-patient case reports, and one was a case series describing four patients, accounting for a total of 27 patients with VEXAS syndrome who had undergone 18-FDG–PET imaging. The main demographic, clinical, genetic features and PET findings of these patients are detailed in Table 1 and are analysed alongside the data from our case series patients in the next paragraph.

**Table 1:** Main clinical and PET imaging characteristics of described cases

Case	Gender and age (years)	UBA1 mutation	Main indications for imaging	Clinical manifestations	18FDG–PET areas of increased intake	References
Described case 1	Male, 80	p.Met41Val	Suspicion for vasculitis	Fever, weight loss, periorbital edema, DVT, anemia, hydrocele, elevated inflammatory markers	Pancreatic isthmus, satellite lymph node, aortic valve	N/A
Described case 2	Male, 85	p.Met41Thr	Fever of unknown origin workup	Relapsing polychondritis, fever, skin vasculitic lesion, and erythema nodosum-like lesions	Bone marrow, nasal and ear cartilage, subclavian origin, lung, spleen	N/A
Described case 3	Male, 75	p.Met41Val	Suspicion for autoinflammatory syndrome	Erythema nodosum, fever, elevated CRP, thrombocytopenia	Bone marrow	N/A
Described case 4	Male, 57	p.Met41Thr	Fever of unknown origin workup	Fever, skin rash, cough, ear chondrites, red eye, asthenia	Lymph nodes	N/A
Described case 5	Male, 85	c.118-1G > C	Fever of unknown origin workup/malignancy suspicion	Fever, arthritis, ear chondritis, asthenia, episode of lower limbs thrombosis	Bone marrow, lung, lymph nodes	N/A
Described case 6	Male, 82	p.Met41Leu	Chronic urticaria, CRP elevation	Fever, chronic urticaria, peripheral neuropathy, arthralgia, arthritis, oedema lower limbs, red eye, ear and nose	Joints, vessels	N/A
Described case 7	Male, 70	p.Met41Leu	Suspicion for adult onset Still disease, Schnitzler syndrome, CRP elevation	Polychondritis, asthenia, peripheral polyneuropathy, recurrent polyserositis, lower limbs thrombosis, arthritis	Bone marrow, muscles	N/A
Described case 8	Male, 51	p.Met41Thr	Unexplained CRP elevation, exclusion of malignancy	Elevated inflammatory markers, DVT and SVT, myelodysplastic syndrome, preseptal eye cellulitis, polychondritis, and panniculitis	Bone marrow, paraortic fibrosis	N/A
Vu KT et al.	Male, 62	p.Met41Thr	Malignancy suspicion	Fever, erythema nodosum, arthritis, periorbital inflammation	Bone marrow, mediastinal and right hilar lymph nodes	[17]
Fagart A et al.	Male, 70	p.Met41Leu	Not reported	Fever, elevated CRP, erythema nodosum	Subcutaneous, cutaneous, and intramuscular, bone marrow, lymph node (axilla, mediastinum, and inguinal), alar cartilage, and nasal septum	[16]
Lohaus A et al.	Male, 77	p.Met41Thr	Workup of autoinflammatory disease	Weight loss, macrocytic anemia, fever, uveitis	Bone marrow, nasal mucosa, cervical lymph nodes, tracheal uptake	[18]
Grambow-Velilla J et al.	Male, 75	p.Met41Thr	Rule out occult malignancy or infection or inflammatory disease	Thrombophlebitis, leukocytoclastic vasculitis, chronic inflammatory arthralgia, and elevated CRP	Thoracic aorta	[19]
van der Made CI et al.	Male, 79	p.Met41Val	Not exactly reported, possibly suspicion of malignancy	Hypertension and nasal polyposis, periorbital and intraorbital swelling, soft tissue infiltration	Bone marrow, mediastinal and hilar lymph nodes, spleen, and thyroid gland	[26]
	Male, 78	p.Met41Val	Not reported	Fatigue, headache, diplopia, night sweats, fever, and elevated inflammatory markers	Bone marrow, spleen, pulmonary nodule, and mediastinal lymph nodes	
	Male, 74	p.Met41Thr	Suspicion for infection or malignancy	Fever, night sweats, fatigue, weight loss, elevated inflammatory markers, and pancytopenia with macrocytic red blood cells	Left ventricles	
	Male, 47	p.Met41Thr	Not reported	Fever, weight loss, night sweats, and erythema nodosum	Bone marrow, lung nodules, pancreas, and parotids	
	Male, 62	p.Met41Thr	Not reported	Elevated inflammatory markers, a normocytic anemia, and thrombocytopenia	PET uptake present, but non-specified	
Midtvedt Ø et al.	Male, “late sixties”	p.Met41Thr	Suspicion vasculitis	Fever, chest pain, fatigue, pulmonary infiltrates, nose chondrites, and elevated CRP	Bone marrow, lung, large vessel (thoracic aorta and aortic arch), and lymph nodes	[20]
Bindoli S et al.	Male, 65	p.Met41Leu	Suspicion of infection	Fever, unilateral pleural effusion, worsening dyspnea, and moderate asthenia	Bone marrow, inferior left lung lobe, and wrist	[6]
Fenu EM et al.	Male, 61	p.Met41Val	Not reported	Fever, disphagia, weight loss, anemia, and DVT	Bone marrow, abdominal lymph nodes, spleen, and submandibular gland	[21]
Ugwoke A et al.	Male, 73	p.Met41Thr	Not reported	Fever, recurrent orbital swelling, thrombophlebitis and weight loss	Bone marrow, and large vessels (ascending aorta, subclavian, carotid arteries)	[22]
Lötscher et al.	Male, 68	p.Met41Thr	Suspicion of malignancy	Polyarthritis	Oesophageal and multiple pulmonary nodules	[23]
Valor-Méndez L et al.	Male, 81	p.Met41Leu	Rule out further foci of inflammation	Erythematous papules, oral ulcerations, painful auricular and nasal erythematous swelling, arthritis in hands and feet, and macrocytic anemia	Bone marrow	[24]
Belicard F et al.	Male, 70	p.Met41Val	Investigate muscle pain	Asthenia, weight loss, palpebral edema, DVT, skin eruption, polychondritis, episcleritis, neurological symptoms, pulmonary infltrates, myalgia	Muscular	[25]
Rubeli S et al.	Male, 60	p.Met41Leu	Suspicion autoinflammatory	Sweet syndrome, arthritis, thromboembolic	Bone marrow	[34]
Lucchino B et al.	Male, 75	p.Met41Val	Not reported	Fever, subcutaneous nodules, anaemia, elevated CRP	Bone marrow and spleen, bilateral lung hypermetabolic consolidation with pleural effusion, latero-cervical lymph nodes, and right spermatic cord	[33]
Austestad J et al.	Male, “sixties"	p.Met41Thr	Rule out inflammatory foci	Fever, abdominal pain, muscular pain, stiffness, and weakness, elevated CRP, thromboembolic events, maculopapular rash	Bone marrow and spleen	[32]
Kunishita Y et al	Male, 66	p.Met41Thr	Not reported	Erythema nodosum, chondritis, fever, myalgia, anaemia, arthritis, headache	Nasal cartilage and bilateral auricular cartilages	[30]
Goyal A et al.	Male, 64	p.Met41Thr	Not reported	Fever, anaemia, elevated CPR, orbital myositis, swelling right leg, right ear, multiple joints	Bone marrow	[31]
Pamies A et al.	Male, 76	p.Met41Thr	Not exactly stated, possibly to investigate persistent CRP elevation	Anaemia, leucopoenia, chondritis, episcleritis, anterior uveitis, dyspnoea, acute renal failure	Bone marrow	[35]
Sakuma M et al.	Male, 61	p.Met41Thr	Myelodysplasia	Anaemia, polychondritis, neutrophilic dermatosis, pneumonia	Bone marrow and pulmonary nodules	[29]
Yildirim F et al.	Male, 67	p.Met41Val	Suspicion for vasculitis	Fatigue, recurrent fever, pulmonary infiltrates, proteinuria, anaemia, leukopenia, increased acute phase reactants, periorbital cellulitis, and SVT	Bone marrow and spleen	[28]
Pozzi M.R.et al.	Male, N/S	p.Met41Val	Not reported	Fever, DVT, PE, arthritis, weight loss, CRP elevation, periorbital oedema, maxillary and ethmoid sinusitis	Bone marrow	[27]
Boret M et al.	Male, 76	p.Met41Val	Workup of FUO	MDS, TIA, fever, night sweat, subcutaneous leg edema, CRP elevation	Bone marrow, legs vascular and muscle tissues, thoracic aorta and the mediastinal adipose tissue	[36]
Fukuda N et al.	Male, 76	p.Met41Thr	Workup of systemic inflammation	Fever, CRP elevation, aseptic peritonitis, headache, abdominal pain, conjunctival hyperaemia, ocular pain, auricular pain, arthralgia, and cutaneous lesions	left superficial temporal artery, nasal and ear cartilages, and lymph nodes (abdominal, axillary, and cervical)	[37]

*UBA1* ubiquitin-like modifier activating enzyme 1, *18-FDG* 18 fluorodeoxyglucose, *PET* positron emitting tomography, *CRP* C-reactive protein, *DVT* deep venous thrombosis, *SVT* superficial venous thrombosis, *N/S* non-specified, *N/A* not allowed, *PE* pulmonary embolism, *FUO* fever unknown origin

### Risk of bias

The retrieved studies were assessed using the JBI tool. Of the 27 retrieved reports, 85.2% were considered “good quality”, and overall, the risk of bias was low, with an average score of 6.1. In particular, 88.9% of the patients had a clear description of the diagnostic tests, which are described in detail in the next paragraph. Given the limited number of available cases, we included all the reports to be as informative as possible and avoid overly restricting the data set, even at the cost of incorporating some lower-quality information. The detailed bias risk assessment is reported in Supplementary Table S1.

### Population characteristics

A total of 35 patients were included in the analysis. All patients were male, with a median age 70 years [interquartile range (IQR) 63.5–76.3 years] (non-specified in three patients). The most common UBA1 mutation was pMet41Thr (51.4%), followed by p.Met41Val (28.6%), and p.Met41Leu (17.1%). One patient had an atypical UBA1 mutation at the splice acceptor site, c.118-2A > G. Clinical manifestations were highly heterogeneous, encompassing almost all the described features of VEXAS syndrome. These data are summarized in Table 1, alongside most relevant PET imaging findings.

### PET imaging analyses

**Fig. 4:**
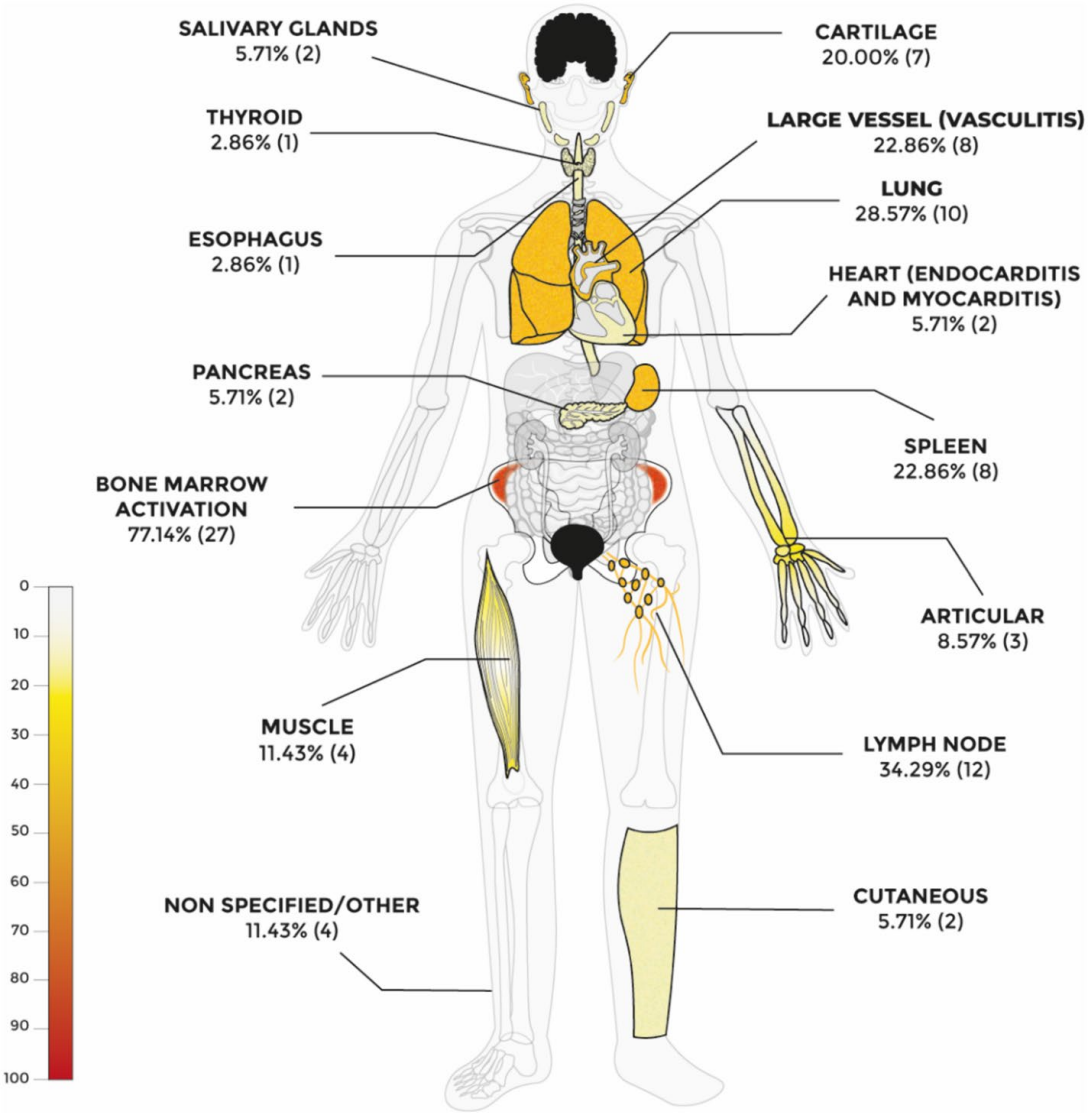
Graphical summary of the involved site at 18-FDG–PET scan. *18-FDG* 18 fluorodeoxyglucose, *PET* positron emitting tomography

Most patients (82.9%) underwent 18-FDG–PET/CT, while the remaining underwent 18-FDG–PET/MR scan. In most patients (37.1%) more than one reason or non-specific indication to perform the PET were reported, as part of the work-up for fever of unknown origin (FUO) or inflammatory markers persistent elevation, and in about one-third of the patients (31.4%) the indication to perform PET was not reported. The most common single reason for performing the imaging was either to rule out malignancies or vasculitis (14.3% each). The identification of infectious foci was also a frequently reported indication, but only in one patient as the only reason to perform PET (2.9%). In some cases, specific clinical manifestations triggered a site-specific investigation, such as to assess muscular, cardiac, or pancreatic involvement [16, 25, 26]. Overall, the sites most frequently showing high metabolic activity at PET scans (graphically summarised in Fig. 4) were bone marrow (77.1%), lymph nodes (34.3%), lungs (28.6%), spleen and large vessels (22.9%), and cartilage (20% each). Less commonly involved organs were the heart, pancreas, salivary glands, skin, muscles and joints, esophagus, and thyroid gland, with most of them described in only one or two patients. Overall, the majority of the patients (71.4%) exhibited multi-organ involvement at PET scan and most lesions were either focal or multifocal, except for diffuse bone marrow hypermetabolism.

Among the eight patients with a large vessel involvement, the most frequent sites of increased uptake were the thoracic aorta (38%), the ascending aorta including the aortic arch (25%), and the subclavian artery (25%), and temporal artery and carotid (13.8%) involvement. In the studied cohort, maximum SUV was reported in six patients (17.6%), with a median SUV value of 6.7 (IQR 6.1–9.4). In addition, six patients (17.1%) had one or more previous PET scans, and these scans were performed an average of 2.7 ± 1.5 years before VEXAS syndrome diagnosis. In two-thirds of these previous scans, a diffuse uptake at the bone marrow level was reported, and in half of them was the only hypermetabolic site. In the other three patients, increased uptake was also observed in lymph nodes, muscles, joints (33.3% each), lungs, and large vessels (16.7% each). Clinical manifestations in these patients were heterogeneous, encompassing constitutional symptoms and CRP elevation (83.3%), skin lesions (urticaria, erythema nodosum, Sweet-like rash) and DVT (50% each), chondritis and periorbital oedema (33.3% each), and anaemia, arthritis, serositis, and polymyalgia rheumatica (16.6% each).

Four patients (11.4%) underwent at least one follow-up PET scan, which took place between 1 and 12 months after the initial scan. For three out of four patients, the scans showed a decrease or a disappearance of the previously identified hypermetabolic areas post-treatment. Conversely, one patient exhibited no changes in PET uptake, despite undergoing glucocorticoid therapy [19].

## Discussion

Our findings suggest that increased ^18^FDG uptake in PET CT/MR scans may be a common finding in patients with VEXAS syndrome. We described 8 patients with VEXAS syndrome with at least one available PET scan, and we retrieved a total of 27 patients from the systematic review of the literature, which account for slightly more than 10% of the described cases [38]. We did not find any specific finding for this condition. The observed PET increased uptakes are rather heterogeneous, and this finding aligns with the known heterogeneity of VEXAS syndrome’s clinical manifestations [39]. Bone marrow hypermetabolism represented the most common site of increased metabolic uptake. This is expected, as bone marrow hypermetabolism is common in patients with anaemia, systemic inflammatory diseases and other autoinflammatory syndromes [40], such as adult-onset Still’s disease (AOSD). AOSD can be considered in the differential diagnosis with VEXAS due to the similarities in several clinical features (e.g., recurrent fever, arthralgia, arthritis, skin rash, and pleuritis) and laboratory markers of inflammation (like high CRP and ferritin). Moreover, areas of high metabolic activity involving spleen, lymph nodes and bone marrow are well-documented in AOSD subjects [41, 42]. Within VEXAS syndrome, increased uptake in bone marrow, and possibly spleen and lymph nodes, is likely tied to systemic inflammation. However, since patients with anaemia and MDS could also display mild bone marrow hypermetabolism [40], more research is required to discern the potential link between bone marrow PET uptake and MDS-related symptoms such as cytopenias and anaemia in VEXAS syndrome. Interestingly, in four patients, bone marrow uptake was increased in PET scans conducted before the clinical onset of the disease. This is consistent with current theories about VEXAS syndrome onset. The disease could either stem from a preclinical, smouldering condition escalating through the clonal expansion of UBA1 mutated myeloid cells or could emerge suddenly from a pre-existing MDS [3, 43]. Clonal expansion in VEXAS is still an expanding topic; however, the hypermetabolic state observed in these cells, which could be detectable by PET imaging, can be attributed to several pathophysiological mechanisms. The UBA1 mutation leads to dysregulation of cellular processes, resulting in an inflammatory microenvironment that promotes myeloid cell proliferation and survival. Specifically, UBA1-mutated myeloid cells exhibit increased activation of innate immune pathways, which may drive their hypermetabolic activity as they respond to chronic inflammatory stimuli. This heightened metabolic state is characterized by enhanced glucose uptake and increased glycolytic activity, as these cells adapt to meet the energy demands of sustained proliferation and inflammatory signalling [44, 45]. Furthermore, co-occurring mutations in genes such as DNMT3A and TET2 may further exacerbate this hypermetabolic phenotype by promoting clonal dominance and skewing differentiation towards myeloid lineages, reinforcing the cycle of inflammation and metabolic activity [8, 46]. Consequently, the hypermetabolic signature observed in PET imaging could reflect the clonal expansion of these mutated cells and serve as a marker of the underlying inflammatory processes that characterize VEXAS syndrome. At the state of the art, however, PET cannot discriminate between pre-existing MDS and UBA1 mutated clones’ expansion without pre-existing MDS, as either scenario could result in similar bone marrow imaging on PET scans conducted years before the diagnosis of VEXAS. In this regard, our findings could serve as an excellent starting point for further in-depth studies into this syndrome’s pathogenesis and differential diagnosis. In addition, given the increased risk of hematological malignancies in patients with VEXAS syndrome [47], PET imaging may be valuable in the follow-up of bone marrow involvement. This technique has proven a useful tool in differentiating between benign and malignant bone marrow conditions [48], suggesting its potential utility in monitoring disease progression in VEXAS patients. Cartilage and skin involvement at PET imaging can be associated with both evident and subclinical chondrites [16, 18], whereas increased ^18^FDG uptake in the lung is ambiguous, making it challenging to differentiate between VEXAS-related manifestations and other conditions such as infectious pneumonia [9]. In assessing the motivations for PET imaging in VEXAS patients, the majority underwent the test for non-specific reasons. However, the need to rule out malignancies, vasculitis, and concealed infectious diseases also frequently drove the decision. Notably, no solid organ malignancy has been reported in any retrieved patient. Possible reasons might be a selection bias, where malignancy cases were simply not described or the absence of biopsy diagnoses. For instance, in our case 1 patient, malignancy in the pancreatic lesion could not be entirely ruled out. Alternatively, VEXAS syndrome patients might just have a low incidence of non-hematological malignancies. However, paraneoplastic syndromes are a potential mimicker of VEXAS syndrome, as they can present, among the others, with constitutional symptoms, skin lesions, cytopenia, and thromboembolic events [49]. Therefore, we maintain that ruling out occult malignancies is among PET's most beneficial roles for patients with consistent elevations in acute phase markers. 18FDG–PET is currently one of the most reliable and convenient imaging techniques in patients suspected of paraneoplastic syndrome, with a sensibility of 81% in detecting underlying malignancies, at least comparable to conventional CT [50, 51]. However, in patients with unexplained systemic inflammation, PET is more versatile because it allows the detection of both malignancies and inflammatory foci, which could go undetected by CT. Furthermore, PET imaging seems useful in uncovering hidden VEXAS manifestations, such as large vessel vasculitis, or cardiac, thyroid, muscular, or pancreatic involvements. These could easily elude detection in routine check-ups. Large vessel vasculitis was observed in roughly a fifth of the patients, a notable percentage considering the seemingly lower prevalence of giant cell arteritis (GCA) and large vessel vasculitis in this syndrome [52]. Concerning with the pancreatic lesion, even without a confirmed pathological diagnosis, the initial reduction of 18-FDG uptake following glucocorticoid therapy suggests a probable inflammatory nature of the lesion, similarly to the finding by Van der Made et al. [26]. Regarding the valvular uptake, given the negative microbiological screenings, imaging, and the initial response to glucocorticoid therapy, we postulate the lesion might signify non-bacterial thrombotic endocarditis (NTBE). While NTBE is generally seen as non-hypermetabolic, it has been observed as hypermetabolic in SLE patients. Hence, PET is not deemed useful in determining the infectious nature of valvular vegetation [53, 54]. To our knowledge, no endocarditis cases or 18FDG uptake at the valvular level have been documented in VEXAS patients, although some instances are reported in relapsing polychondritis [55]. Most hypermetabolic lesions in VEXAS patients, NTBE included, might stem from the accumulation of neutrophils and white blood cells, a theory backed by findings in skin lesions [56, 57], and possibly in the lungs, even if lung pathology is primarily derived from alveolar lavage data [9]. The hypermetabolic nature of NTBE in VEXAS syndrome might originate from valvular cartilage inflammation, leading to neutrophil accumulation. Similarly, widespread muscular uptake also bears resemblance to sarcoidosis' “leopard man” and multifocal myositis, thus adding them to the list of possible differential diagnoses for VEXAS syndrome [16, 58, 59]. Of note, despite its frequency in patients with VEXAS syndrome [60], any patient in our study displayed periorbital uptake. The present study has some limitations. The limited number of reported cases challenges firm conclusions about the prevalence of PET findings, particularly for rarer manifestations. In addition, many reports lack specific data about PET imaging, notably missing SUV values, a significant aspect of PET imaging in VEXAS syndrome. Another potential bias is the omission of negative findings—cases where PET imaging yielded no anomalies and thus went unreported. Lastly, we have limited data about follow-up PET scans and concomitant clinical manifestations, leaving the potential role of PET in VEXAS syndrome monitoring an unresolved issue. We encourage further research to correlate imaging results with clinical and pathogenic factors, especially when based on biopsy-derived pathological data. In addition, more longitudinal data involving pre-clinical bone marrow uptake and the variation of SUV after treatment would help improve our understanding of the disease's natural evolution and the response to treatment.

## Conclusions

The predominant PET scan finding was bone marrow hypermetabolism, which might predate the clinical onset of the disease. The primary utility of PET imaging in VEXAS syndrome is to rule out concomitant malignancies and to detect non-clinically evident manifestations, such as large vessel involvement. However, it does not appear to be helpful from a diagnostic perspective, due to the non-specific nature of findings and its inability to distinguish between infective or inflammatory origins, especially in lung and cardiac findings. Occurrences such as endocarditis and pancreatic nodules need further investigation before they can be definitively attributed to VEXAS syndrome. Additional studies are required to determine the role of PET in follow-up and to describe the imaging findings more accurately.

## Supplementary Information

Below is the link to the electronic supplementary material.

Supplementary file1 (DOCX 621 KB)

Supplementary file2 (PDF 86 KB)

## Data Availability

No additional data are available.

## Declarations

### Patient and public involvement statement

This research was done without direct patient involvement. Patients were not invited to comment on the study design and were not consulted to develop patient-relevant outcomes or interpret the results. Patients were not invited to contribute to the writing or editing of this document for readability or accuracy.
