# Supplementary material for: The role of 18FDG–PET imaging in VEXAS syndrome: a multicentric case series and a systematic review of the literature
Source: Intern Emerg Med. 2024 Sep 9;19(8):2331–45. doi: 10.1007/s11739-024-03763-9 (PMC11582098; doi:10.1007/s11739-024-03763-9)
Supplement: Supplementary file 1 — Supplementary file1 (DOCX 621 KB) [file 11739_2024_3763_MOESM1_ESM.docx]

**Supplementary Material**

**Supplementary Data S1. Literature search strategies**

The query was performed on MedLine via Pubmed (from 1946 to week 2 January 2024), EMBASE via OVID (from 1974 to 17.01.24), and Cochrane Library via Cochrane Central (to 17.01.2024), using the following MESH terms: VEXAS.mp.; Ubiquitin-Activating Enzymes.mp. ; Hereditary Autoinflammatory Diseases.mp.; positron emission tomography/ or positron emission tomography-computed tomography/ or fluorodeoxyglucose f 18/.

The following search strategies have been implemented:

[1] VEXAS.mp.
[2] Ubiquitin-Activating Enzymes.mp.
[3] Hereditary Autoinflammatory Diseases.mp.
[4] positron emission tomography/ or positron emission tomography-computed tomography/ or fluorodeoxyglucose f 18/
[5] 1 or 2 or 3 
[6] 4 and 5

**Supplementary figure S1. Flowchart of the literature research**


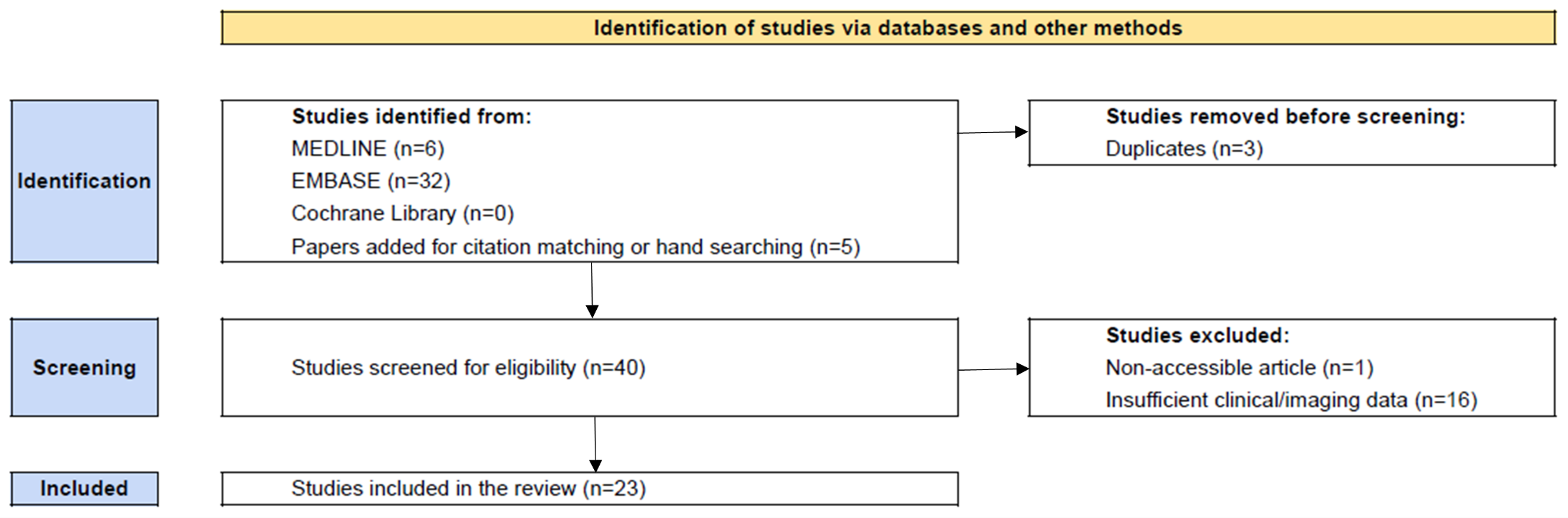


**Supplementary Table S1. Risk of bias assessment**

| **Study** | **1. Were patient’s demographic characteristics clearly described?** | **2. Was the patient’s history clearly described and presented as a timeline?** | **3. Was the current clinical condition of the patient on presentation clearly described?** | **4. Were diagnostic tests or assessment methods and the results clearly described?** | **5. Was the intervention(s) or treatment procedure(s) clearly described?** | **6. Was the post-intervention clinical condition clearly described?** | **7. Were adverse events (harms) or unanticipated events identified and described?** | **8. Does the case report provide takeaway lessons?** | **Total score** |
| --- | --- | --- | --- | --- | --- | --- | --- | --- | --- |
| Vu T et al. | yes | yes | yes | yes | yes | yes | unclear | yes | 7 |
| Fagart A et al. | yes | no | no | yes | no | no | no | yes | 3 |
| Lohaus N et al. | yes | no | no | yes | no | no | no | yes | 3 |
| Grambow-Velilla et al. | yes | no | no | yes | no | no | no | yes | 3 |
| Van der Made CI et al. | yes | yes | yes | yes | yes | yes | unclear | yes | 7 |
| Van der Made CI et al. | yes | yes | yes | yes | yes | yes | unclear | yes | 7 |
| Van der Made CI et al. | yes | yes | yes | yes | yes | yes | unclear | yes | 7 |
| Van der Made CI et al. | yes | yes | yes | yes | yes | yes | unclear | yes | 7 |
| Van der Made CI et al. | yes | yes | yes | yes | yes | yes | unclear | yes | 7 |
| Midtvedt Ø et al. | unclear | yes | yes | yes | yes | unclear | no | no | 4 |
| Bindoli S et al. | yes | yes | yes | yes | yes | yes | yes | yes | 8 |
| Fenu EM et al. | yes | no | no | yes | no | no | no | yes | 3 |
| Ugwoke A et al. | yes | yes | yes | yes | yes | yes | yes | no | 7 |
| Lötscher F et al | yes | yes | yes | yes | yes | yes | yes | yes | 8 |
| Valor-Méndez et al. | yes | yes | yes | yes | yes | yes | yes | yes | 8 |
| Belicard F et al. | yes | yes | yes | yes | yes | yes | yes | yes | 8 |
| Rubeli S et al. | yes | yes | yes | unclear | yes | yes | unclear | no | 5 |
| Lucchino B et al. | yes | unclear | yes | yes | yes | yes | no | yes | 6 |
| Austestad J et al. | unclear | yes | yes | yes | yes | yes | no | no | 6 |
| Kunishita Y et al. | yes | yes | yes | yes | yes | yes | yes | yes | 8 |
| Goyal A et al. | yes | yes | yes | yes | yes | yes | unclear | yes | 7 |
| Pamies A et al. | yes | yes | yes | yes | yes | unclear | unclear | unclear | 5 |
| Sakuma M et al. | yes | yes | yes | yes | yes | yes | yes | yes | 8 |
| Yildrim F et al. | yes | yes | yes | yes | yes | yes | no | yes | 7 |
| Pozzi MR et al. | unclear | yes | yes | unclear | yes | yes | no | unclear | 4 |
| Boret M et al. | yes | unclear | yes | unclear | yes | yes | no | unclear | 4 |
| Fukuda et al. | yes | yes | yes | yes | yes | yes | yes | yes | 8 |
